# Genistein during Development Alters Differentially the Expression of POMC in Male and Female Rats

**DOI:** 10.3390/metabo11050293

**Published:** 2021-05-02

**Authors:** Jose Manuel Fernandez-Garcia, Beatriz Carrillo, Patricia Tezanos, Paloma Collado, Helena Pinos

**Affiliations:** 1 Departamento de Psicobiología, Facultad Psicología, Universidad Nacional de Educación a Distancia (UNED), 28040 Madrid, Spain; jmferdande@psi.uned.es (J.M.F.-G.); bcarrillo@psi.uned.es (B.C.); pcollado@psi.uned.es (P.C.); 2 Instituto Mixto de Investigación Escuela Nacional de Sanidad-UNED (IMIENS), 28040 Madrid, Spain; 3 Departamento de Neurociencia Traslacional, Instituto Cajal, Consejo Superior de Investigaciones Científicas (CSIC), 28002 Madrid, Spain; ptezanos@cajal.csic.es

**Keywords:** genistein, proopiomelanocortin, arcuate nucleus, sex differences, rats

## Abstract

Phytoestrogens are considered beneficial for health, but some studies have shown that they may cause adverse effects. This study investigated the effects of genistein administration during the second week of life on energy metabolism and on the circuits regulating food intake. Two different genistein doses, 10 or 50 µg/g, were administered to male and female rats from postnatal day (P) 6 to P13. Physiological parameters, such as body weight and caloric intake, were then analyzed at P90. Moreover, proopiomelanocortin (POMC) expression in the arcuate nucleus (Arc) and orexin expression in the dorsomedial hypothalamus (DMH), perifornical area (PF) and lateral hypothalamus (LH) were studied. Our results showed a delay in the emergence of sex differences in the body weight in the groups with higher genistein doses. Furthermore, a significant decrease in the number of POMC-immunoreactive (POMC-ir) cells in the Arc in the two groups of females treated with genistein was observed. In contrast, no alteration in orexin expression was detected in any of the structures analyzed in either males or females. In conclusion, genistein can modulate estradiol’s programming actions on the hypothalamic feeding circuits differentially in male and female rats during development.

## 1. Introduction

Genistein is a phytoestrogen that belongs to the group of isoflavones. It is present in a wide variety of legumes, mainly soybeans and their derivatives, which makes it one of the most consumed phytoestrogens by humans [1]. Soy is a typical ingredient in the traditional Asian diet, and it is also a widely consumed food in Western countries, being one of the most common milk substitutes, mostly in children [2,3,4].

Phytoestrogens exert their actions principally through the estradiol receptor (ER) α and ERβ, with higher reported affinity to the latter [5,6,7], but also through the G protein-coupled estrogen receptor (GPER) [8,9]. Specifically, genistein has structural similarities with estradiol, which allows it to bind estrogen receptors, acting as a potential agonist or antagonist of the estrogens, depending on the estradiol levels or the tissue [10,11,12,13,14].

Phytoestrogens are considered endocrine disruptors (EDCs), and although most of the EDCs have been demonstrated to have harmful effects on the organism (e.g., pesticides, bisphenol A) [15], phytoestrogens, in general, seem to have beneficial effects on health. Some practical advantages include the prevention of cardiovascular diseases [16], decreased inflammatory response in microglia [17], prevention of denervation-induced muscle atrophy [18], prevention of different types of cancer [19,20] and improvement in menopausal symptoms [21,22]. However, not all results evidence beneficial actions on the organism due to reports of damaging effects arising from phytoestrogens′ exposure. In addition, a proportion of clinical studies do not demonstrate a clear health improvement (for review, see [23,24,25]).

The basal sexual genetic differences have an impact on structural, metabolic and behavioral differences between male and female rats that are more evident in adulthood [26,27,28]. Some authors have recently shown that genistein produces some alterations in various neural systems, such as vasopressinergic or dopaminergic systems, mainly when administered during development, and that those effects are sexually dimorphic in males and females [29,30]. Moreover, previous results of our group have demonstrated that estradiol administered from postnatal day (P) 6 to P13 has a modulatory role in rats in the early stages of life due to under- or overnutrition [31,32,33], specifically in the programming of the body weight in males and the mRNA POMC hypothalamic levels in females [34]. Estradiol conveyed mainly through ERα [35,36,37,38,39] is involved in the regulation of energy metabolism inhibiting food intake [40]. Considering these results and the possible estrogenic/antiestrogenic effects of genistein, it seems reasonable to assume that exposure to genistein during the early postnatal period may produce some alteration to the development of energy metabolism and on hypothalamic circuits that regulate food intake. Numerous orexigenic and anorexigenic peptides are involved in the regulation of body weight and feeding. Among them are the orexin and POMC peptides. The former are synthesized and released from LH neurons and increase food intake in response to the release of neuropeptide Y (NPY) from Arc neurons [41,42]. The latter, anorexigenic in their activity, are expressed by Arc neurons that send satiety signals to the LH and paraventricular hypothalamic nuclei (PVH) [43].

Given the increase in soy consumption in the general population and in children in particular, it is necessary to determine the effects of its main component, genistein. In the present work, we analyzed genistein treatment’s effect during the second week of life on physiological parameters such as body weight and food consumption and neurohormonal parameters, such as POMC and orexin hypothalamic expression, in male and female rats.

## 2. Results

### 2.1. Differences in Body Weight and Caloric Intake

In the evolution of body weight, a main effect of sex (F1,50 = 253.559; *p* < 0.001) was found. Neither the treatment (F2,50 = 0.170; *p* = 0.844) nor the interaction (F2,50 = 0.520; *p* = 0.598) showed a significant effect.

**Figure 1:**
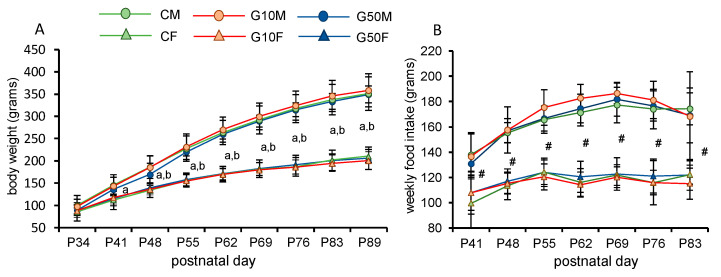
(**A**) Body weight evolution in all groups. (**B**) Weekly food intake in all groups (repeated measured ANOVA). Statistically significant differences (*p* < 0.05) are labelled as follows: a = sex differences in C and G10 groups; b = sex differences in G50 groups. # = sex differences in all groups studied. All values are expressed as means ± S.D. CM: control males: CF: control females; G10M: genistein treated males, dose 10 µg/g; G10F: genistein treated females, dose 10 µg/g; G50M: genistein treated males, dose 50 µg/g; G50F: genistein treated females, dose 50 µg/g.

As shown in Figure 1A, sex differences in body weight appeared on P41 in control and G10 groups (*p* < 0.05 in all cases), with males heavier than females. A delay in the appearance of sex differences can be observed in G50 groups from P48 onwards (*p* < 0.05 in all cases). No significant differences were observed when males and females were analyzed separately.

Food intake was measured in grams. A main effect of sex (F1,50 = 297.788; *p* < 0.001) was found. Treatment (F2,50 = 0.183; *p* = 0.833) and an interaction between these two factors (F2,50 = 0.696; *p* = 0.504) were not significant. As can be seen in Figure 1B, sex differences appeared on P41 and continued until P83. In all groups, males ate more than the corresponding females.

### 2.2. Orexin-ir and POMC-ir Cell Analysis

**Figure 2:**
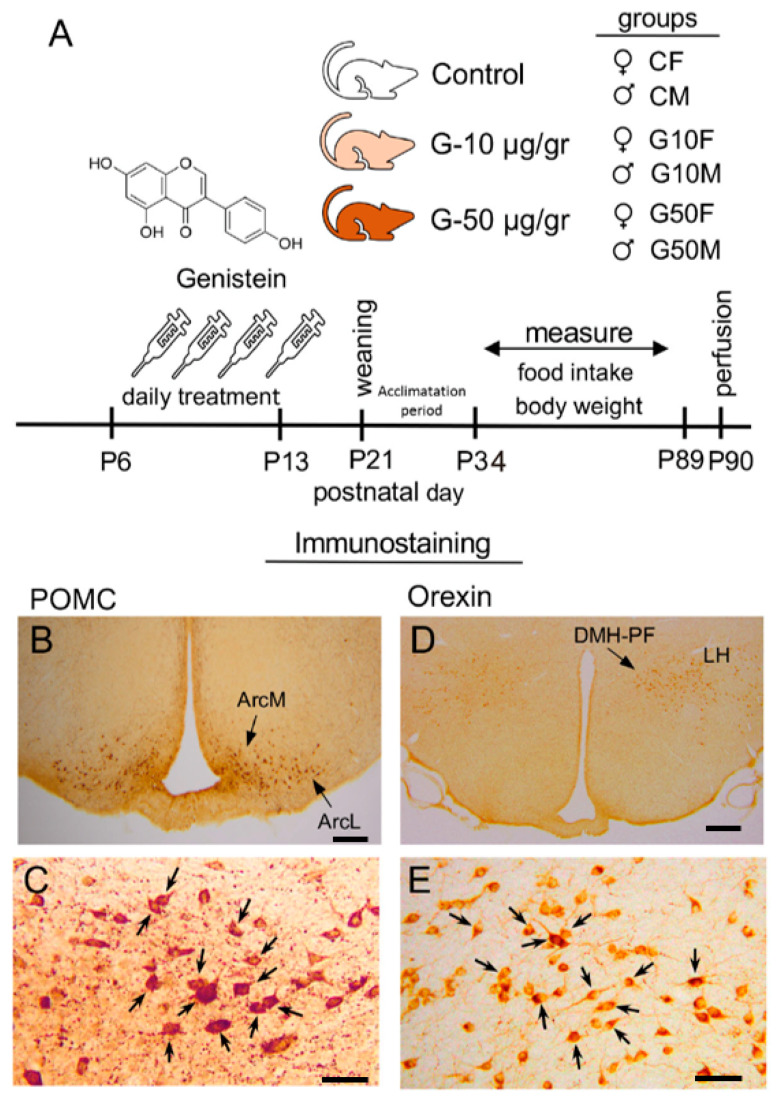
(**A**) Schematic representation of the procedure used from treatment to immunostaining. From P6 to P13, daily injections of synthetic genistein (10 or 50 µg/g) or vehicle according to experimental group were administered. From weaning on P21 to P34, an acclimatization period was implemented. Food intake and body weight were measured weekly from P34 until P89. Animals were sacrificed on P90. (**B**,**C**) Photomicrographs showing the distribution of immunostaining of POMC-ir positive cells in Arc nucleus. (**D**,**E**) Orexin-ir positive cells in the PF and LH. Arrows show orexin-ir and POMC-ir positive cells counted. ArcM: arcuate medial subdivision, ArcL: arcuate lateral subdivision; LH: lateral nucleus of the hypothalamus; DMH-PF: dorsomedial-perifornical nucleus B Bar = 200 μm; C Bar = 50 μm ; D Bar = 300 μm; E Bar = 75 μm [44].

No orexin-ir cells were detected in the ventromedial (VMH) or paraventricular hypothalamic nuclei (PVH). Orexin-ir cells were observed in the medial-ventral area of the lateral hypothalamus (LHmv). Likewise, a small population of orexin-ir cells was detected in the lateral edge of the dorsomedial hypothalamic nucleus (DMH) adjacent to the orexin-ir cells in the perifornical nucleus (PF). Together, these two areas were considered as the DMH-PF continuum for counting and analysis. In all nuclei studied, the cells that expressed orexin were easily detectable because the cell body was heavily labelled (Figure 2D,E).

No differences between the hemispheres were found in the PF-DMH continuum (F1,54 = 0.926; *p* = 0.402) or the LH (F1,54 = 1.196; *p* = 0.310). Moreover, no main effect of sex (F1,30 = 0.598; *p* = 0.445), treatment (F2,30 = 1.614; *p* = 0.216) or interaction between the factors (F2,30 = 0.079; *p* = 0.924) was found in this same continuum, and similar results were also observed in the LH with no main effect of sex (F1,30 = 0.002; *p* = 0.964), treatment (F2,30 = 0.218; *p* = 0.805) or interaction between the factors (F2,30 = 0.234; *p* = 0.793).

POMC-ir cells were easily distinguishable because the cell body was heavily labelled (Figure 2B,C). Cells expressing POMC were detected in the medial (ArcM), lateral (ArcL) and posteromedial (ArcPM) subdivisions of the arcuate nucleus (Arc) but not in the ArcDorsal subdivision.

No differences between the hemispheres were found in the anterior arcuate (F1,56 = 0.001; *p* = 0.979) or the posterior arcuate (F1,56 = 0.021; *p* = 0.884).

In the ArcM, a main effect of sex was found (F1,36 = 5.347; *p* < 0.001) but no effect of treatment (F2,36 = 0.196; *p* = 0.823) or interaction (F2,36 = 1.051; *p* = 0.164). Moreover, in the ArcL and the ArcPM, no effect of sex (F1,36 = 0.001; *p* = 0.973; F1,36 = 0.795; *p* = 0.379, respectively), treatment (F2,36 = 1.514; *p* = 0.234; F2,36 = 0.724; *p* = 0.492, respectively) or interaction (F2,36 = 1.905; *p* = 0.164; F1,36 = 0.467; *p* = 0.631, respectively) were found.

**Figure 3:**
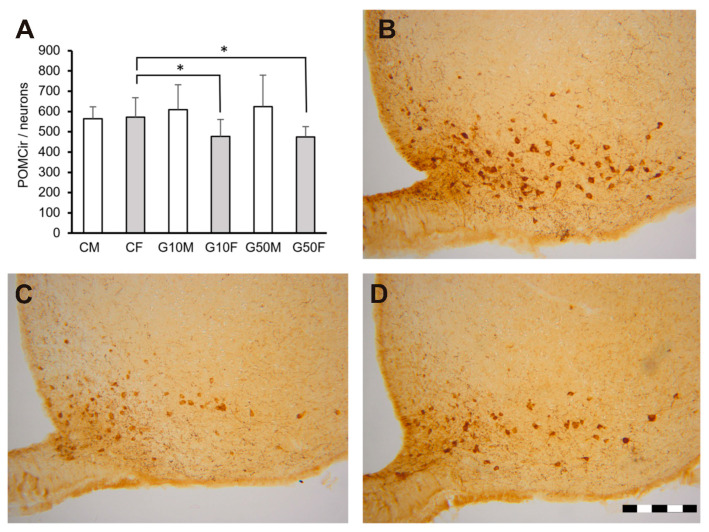
Graph (**A**) showing the number of POMC-ir neurons in ArcM in all experimental groups (two-way ANOVA) * indicates differences between groups (*p* < 0.05 in all cases). The error bars indicate standard error of the mean. CM: control males; CF: control females; G10M: genistein treated males, dose 10 µg/g; G10F: genistein treated females, dose 10 µg/g; G50M: genistein treated males, dose 50 µg/g; G50F: genistein treated females, dose 50 µg/g. *n* = 6 in all groups. All treatments, either injection of genistein or vehicle, were administered from P6 to P13. (**B**–**D**) photomicrographs showing the distribution of POMC-ir positive cells in Arc nucleus. **B** = control female, **C** = G10 female, **D** = G50 female. Bar = 200 μm [44].

Analysis of each sex separately showed a difference in the ArcM in females. The CF exhibited a greater number of POMC-ir cells than the females treated with low or high genistein doses (*p* < 0.05, in both cases). In contrast, males did not show significant differences among the three groups studied (Figure 3).

## 3. Discussion

The present study results showed that the exposure to genistein in the early stages of development modifies hypothalamic POMC neurons’ long-term expression in the arcuate nucleus of female but not male Wistar rats. Moreover, high doses of genistein produced a delay in the emergence of sex differences in body weight. In contrast, caloric intake or orexin expression were not altered in either sex.

Treatment with genistein from P6 to P13 did not affect the caloric intake because there were no differences between the groups or both sexes’ developmental pattern. The differences appeared from P48 onwards in all groups. Concerning the body weight, we detected differences in the evolution of normal sexual dimorphism, an observation resulting from the one week delay in the emergence of sex differences in the genistein groups with high dose. Sex differences in control and G10 groups were observed from P41 onwards but at P48 in G50 groups. Similar results in the emergence of sex differences in control groups were reported in a previous study by our group [32].

Although the effects of genistein during development do not significantly alter physiological parameters such as caloric intake or body weight, a relevant effect has been detected in the brain. Specifically, the number of cells expressing POMC decreased in the medial subdivision of the arcuate nucleus of female rats when low or high doses of genistein were administered from P6 to P13. However, no effect of genistein was detected on POMC expression in this same nucleus in males.

The fact that genistein treatment altered the expression of POMC in the ArcM in female but not male rats is not surprising if previous results from our group are considered. In the same postnatal period, the administration of estradiol modulated the levels of hypothalamic POMC mRNA in females on a low-protein or high-fat diet, but no effect was detected in the male rats or in any other feeding-related peptide studied [31,32]. Furthermore, when the activity of ERα, ERβ and GPER receptors was blocked from P5 to P13, there was a decrease in hypothalamic POMC mRNA levels in female rats, but again, this effect was not detected in males, and no other alterations were shown in other peptides studied [34]. In line with these data, the results of the present study show first that genistein through an agonist or antagonist estrogenic activity alters the long-term expression of POMC during development in female but not male rats, and therefore this phytoestrogen could interfere with the programming activity of estradiol early in life. Secondly, in all cases, a misbalance in estrogenic activity affects POMC but no other peptides related to food intake in the hypothalamus [31,32] or specifically orexin in the DMH, PF or LH nuclei.

Gao et al. [45] demonstrated the existence of a direct relation between estradiol and POMC since this hormone can increase excitatory inputs on POMC neurons and POMC tone in the Arc in rats and mice [45]. This fact can explain the consistent response of POMC to the changes in the estradiol activity during development and how important the activity of this hormone is to the long-term expression of POMC in the hypothalamus. Our results show that the influence of genistein on estrogenic activity during development also results in an alteration of the melanocortin system in females in the long term.

At this point, it is important to note that the administration of estradiol in control animals in the same postnatal period did not alter hypothalamic POMC mRNA levels either in males or females in adulthood [32], suggesting a specific antagonist effect of genistein on the activity of estradiol in the programming of feeding circuits during development. On the one hand, it is worth bearing in mind that the effects of estradiol on food intake are mainly via the ERα [35,36,37,38,39] and that genistein has a higher affinity for ERβ, which could lead to differential effects of the phytoestrogen compared to estradiol. To our knowledge, no direct effect of genistein on POMC has been reported, but some results suggest a possible indirect effect of genistein on the downregulation of POMC expression in the Arc. It has been demonstrated that dietary soy produces an increase in hypothalamic neuropeptide Y (NPY) or agouti-related protein (AgRP) levels [46,47,48] and the inhibitory action of NPY/AgRP neurons on POMC neurons, possibly through GABA, has been soundly demonstrated [49,50,51,52,53]. Further research is therefore needed to determine the specific action of genistein during the programming period on ERs and whether genistein acts directly on POMC neurons or whether its effects on this system are due to an indirect action via NPY.

Numerous studies have reported that soy proteins may exert beneficial health effects by improving metabolic parameters and preventing obesity and diabetes, mainly in adult pre-and postmenopausal women [54,55,56]. However, many studies have shown that exposure to phytoestrogens early in development has adverse effects on reproductive function (see [57], for review) and alters various neurotransmitter systems [29,30,58]. These data, along with the results of the present work, demonstrate that exposure to genistein during the most sensitive stages of development alters neurotransmitter and neuropeptidergic systems involved in reproductive or feeding neurohormonal systems.

Very few studies have paid attention to the effects of phytoestrogens during development on the functions of the hypothalamic circuits regulating energy metabolism and/or feeding disorders. Data from other authors reported that phytoestrogens alter the reproductive system [57], and now our results reveal that phytoestrogens can also differentially modulate some actions of the estradiol during development in male and female rats. Taking into account that soy is the main substitute food for milk in children and that the lactation period is a sensitive period for the optimal development of brain circuits, further research will be needed to unravel the mechanisms through which genistein during development may alter the intake system in order to detect adverse effects on energy metabolism and feeding.

## 4. Materials and Methods

### 4.1. Animals

All experiments were designed according to the guidelines published in the “NIH Guide for the care and use of laboratory animals”, the principles presented in the “Guidelines for the Use of Animals in Neuroscience Research” by the Society for Neuroscience, the European Union legislation (Council Directives 86/609/EEC and 2010/63/UE) and the Spanish Government Directive (R.D. 1201/2005). Experimental procedures were approved by our Institutional Bioethical Committee (UNED, Madrid). Special care was taken to minimize animal suffering and reduce the number of animals used to the minimum necessary. Wistar rats were reared under stable temperature, humidity and light conditions (22 ± 2 °C; 55 ± 10% humidity; 12 h light/12 h dark cycle, lights on from 08:00 to 20:00) with food and water ad libitum. For mating, a male was placed in a cage with two females for one week. Pregnant females were housed individually in plastic maternity cages with wood shavings as the nesting material. On postnatal day 1 (P1), pups born on the same day were weighed, sexed, and randomly distributed (five females and five males/dam). From P6 to P13, pups were treated with a daily s.c. injection of vehicle (corn oil), or synthetic genistein (Genistein Synthetic, ≥98%, Sigma-Aldrich St. Luois, MO, USA) in two doses: a low dose of genistein (10 µg/g) or a high dose of genistein 50 µg/g. The 10 µg dose was previously used by other authors who obtained differential effects in males and females on several physiological and brain parameters [59,60,61] This experimental design resulted in the following groups: control male (*n* = 10, CM), control female (*n* = 9, CF), G10 male (*n* = 10, G10M), G10 female (*n* = 9, G10F), G50 male (*n* = 10, G50M) and G50 female (*n* = 8, G50F). *n* = 6 and *n* = 7 in each group were used to study orexin and POMC expression, respectively. From weaning on P21 to P34, an acclimatization period was implemented. From P33 to P89, body weight and food intake were measured every 7 days, except for the week prior to perfusion when body weight was recorded at 6 days (Figure 2A).

### 4.2. Tissue Preparation

On P90, animals were deeply anaesthetized with an overdose of tribromoethanol in saline (1 mL/kg). Then, the animals were transcardially perfused with saline followed by 4% paraformaldehyde (PAF). The brains were removed, stored in a freshly prepared PAF solution for two hours at 4 °C and then washed several times in phosphate-buffered saline (PBS). Next, the brains were stored in a 30% sucrose solution in PBS at 4 °C until they were examined. The brains were then frozen on dry ice and serially sectioned along the coronal plane at a thickness of 40 μm. Serial sections were collected in four series, two of which were used in this study processed as free-floating sections for orexin and POMC immunostaining.

### 4.3. Orexin and POMC Immunostaining

The sections were incubated in PBS overnight. Endogenous peroxidase activity was blocked by incubation with H_2_O_2_ in 0.5% Triton X-100 in PBS for 30 min. After a brief wash in PBS, the sections were incubated in normal goat serum (diluted 1:5 in PBS; Vector, Burlingame, CA, USA) for 30 min at room temperature. Then, the sections were incubated for 48 h at 4 °C in a rabbit anti-orexin A primary antibody (Calbiochem, San Diego, CA, USA) or in a rabbit anti-POMC primary antibody (Phoenix Pharmaceuticals Inc., Burlingame, CA, USA); 1:2000 in both cases. After several brief washes in PBS, the sections were incubated with biotinylated anti-rabbit IgG serum (Vector, 1:200) for 90 min and then in avidin-peroxidase complex (Immunopure ABC Vector Burlingame, CA, USA) for 60 min at room temperature. Finally, the presence of peroxidase activity was visualized with a solution containing 0.02 g/mL diaminobenzidine (DAB; Aldrich, Madrid, Spain) and 0.025% hydrogen peroxidase in Tris–HCl, pH 7.6. The sections were mounted on gelatin-coated slides, dehydrated in ethanol, washed in xylene and coverslipped with DPX (Surgipath Europe Ltd., Peterborough, UK).

The number of orexin-ir cells in the dorsomedial hypothalamus (DMH), perifornical area (PF) and lateral hypothalamus (LH) and the number of POMC-ir cells in the subdivisions of the arcuate nucleus (the dorsal [ArcD], medial [ArcM], lateral [ArcL] and medial posterior [ArcPM]) [44] were estimated. Briefly, a microphotograph of each section was acquired using a scanner (Nikon Collscope Eclipse Net-VSL, Tokyo, Japan) with a monitor (Digital Sight DS-L1, Tokyo, Japan). The number of orexin-ir or POMC-ir cells in each section was estimated using ImageJ (ImageJ bundled with 64-bit Java 1.8.0; National Institutes of Health, Bethesda, MD, USA) following the Königsmark cell counting procedure [62]. The scattered orexin-ir cells on the lateral edge of the DMH were considered to be continuous with the PF nucleus. The orexin-ir or POMC-ir cells included within the boundaries of the different nuclei were counted.

### 4.4. Statistical Analysis

The evolution of body weight and caloric intake during the experimental procedure was analyzed using repeated-measures ANOVA with treatment as the within-subject factor and body weight and caloric intake as the between-subject factors. To determine the differences among the groups, one-way ANOVA was performed when appropriate. Post hoc comparisons were performed with Student–Newman–Keuls tests. The significance level was set at *p* < 0.05.

The number of orexin-ir and POMC-ir cells in both hemispheres was estimated. The data were subjected to one-way ANOVA with the hemisphere as a factor to determine the potential differences between the right and left hemispheres. Once the effect of the hemisphere was discarded, the mean value of the two hemispheres was used for statistical analysis performed by one-way ANOVA followed by Student–Newman–Keuls tests when appropriate, and the significance level was *p* < 0.05.

## Data Availability

The data presented in this study are available on request from the corresponding author.
